# Supplementary material for: MTMR7 regulates human spermatogonial stem cells proliferation and migration via targeting FLNB
Source: PLoS One. 2025 Jul 10;20(7):e0327669. doi: 10.1371/journal.pone.0327669 (PMC12244780; doi:10.1371/journal.pone.0327669)
Supplement: S2 Table — (DOCX) [file pone.0327669.s002.docx]

| **Class** | **Receptor position** | **Receptor residue** | **Ligand position** | **Ligand residue** | **Interaction distance** |
| --- | --- | --- | --- | --- | --- |
| Hydrophobic Interactions | E28 | GLU | T491 | THR | 3.9 |
| Hydrophobic Interactions | V56 | VAL | F495 | PHE | 3.41 |
| Hydrophobic Interactions | L57 | LEU | F495 | PHE | 3.78 |
| Hydrophobic Interactions | R61 | ARG | Q515 | GLN | 3.64 |
| Hydrophobic Interactions | K65 | LYS | R514 | ARG | 3.26 |
| Hydrophobic Interactions | K65 | LYS | Y520 | TYR | 2.52 |
| Hydrophobic Interactions | Q68 | GLN | V517 | VAL | 3.95 |
| Hydrophobic Interactions | P212 | PRO | T491 | THR | 3.28 |
| Hydrophobic Interactions | Q743 | GLN | L535 | LEU | 3.73 |
| Hydrogen Bonds | K31 | LYS | T491 | THR | 3.09 |
| Hydrogen Bonds | Q59 | GLN | F499 | PHE | 1.78 |
| Hydrogen Bonds | K60 | LYS | M297 | MET | 2.82 |
| Hydrogen Bonds | R61 | ARG | Q515 | GLN | 2.94 |
| Hydrogen Bonds | M62 | MET | Q515 | GLN | 3.23 |
| Hydrogen Bonds | V127 | VAL | E305 | GLU | 2.87 |

**List of  interaction pairs between FLNB and MTMR7**
