# Supplementary material for: MTMR7 regulates human spermatogonial stem cells proliferation and migration via targeting FLNB
Source: PLoS One. 2025 Jul 10;20(7):e0327669. doi: 10.1371/journal.pone.0327669 (PMC12244780; doi:10.1371/journal.pone.0327669)

Related to Fig 1B

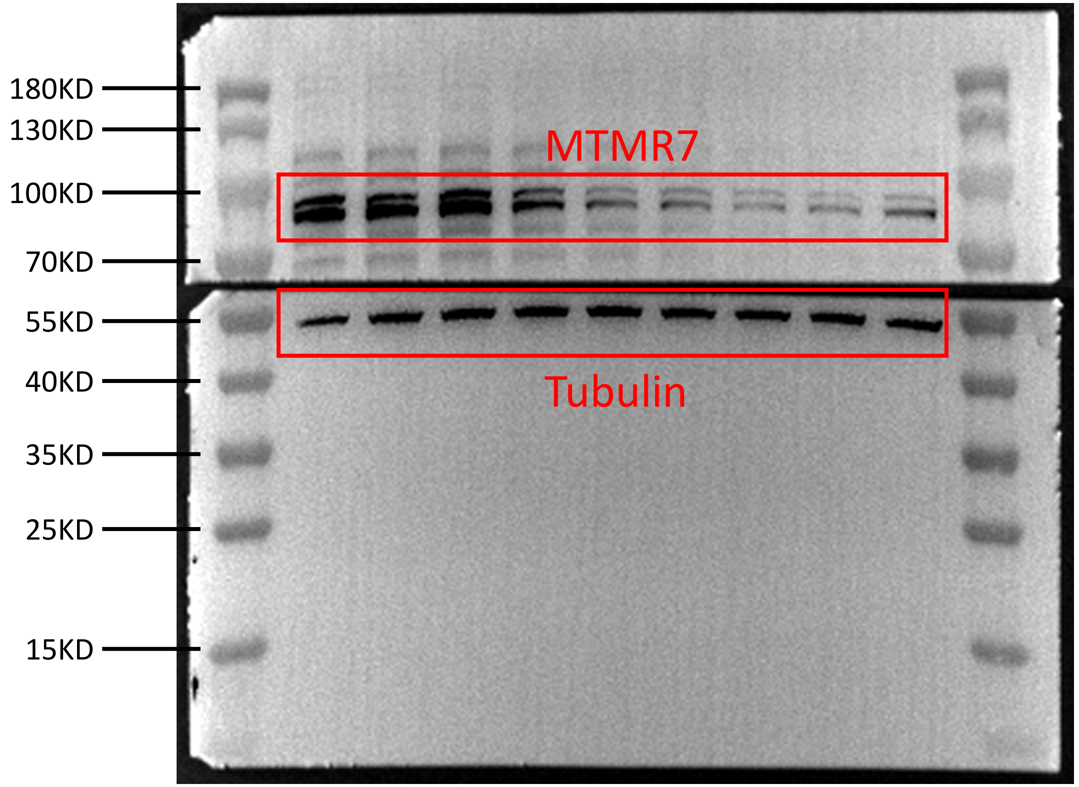

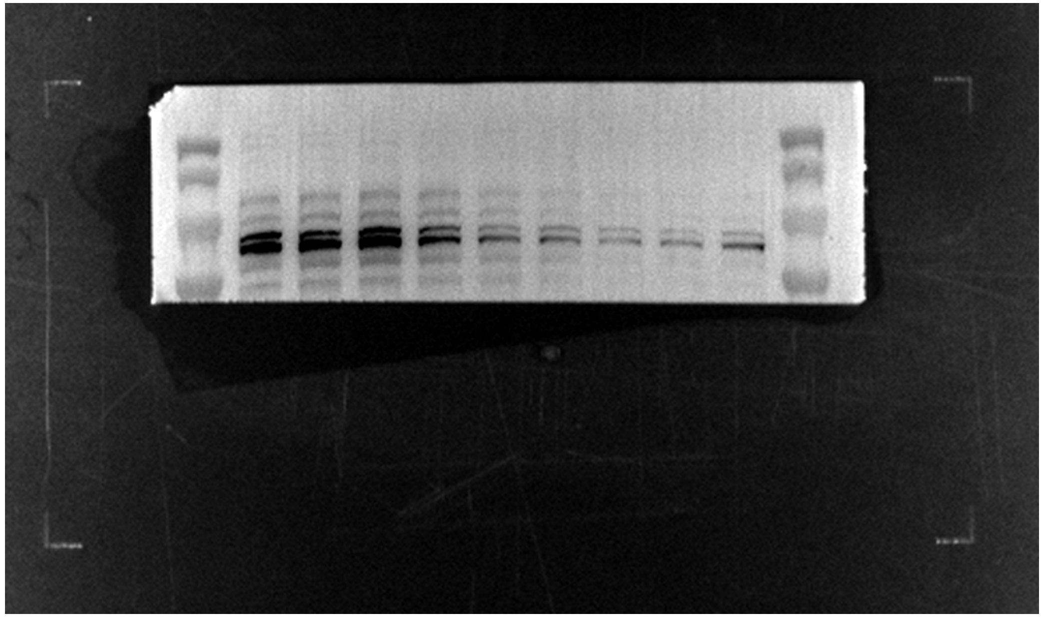

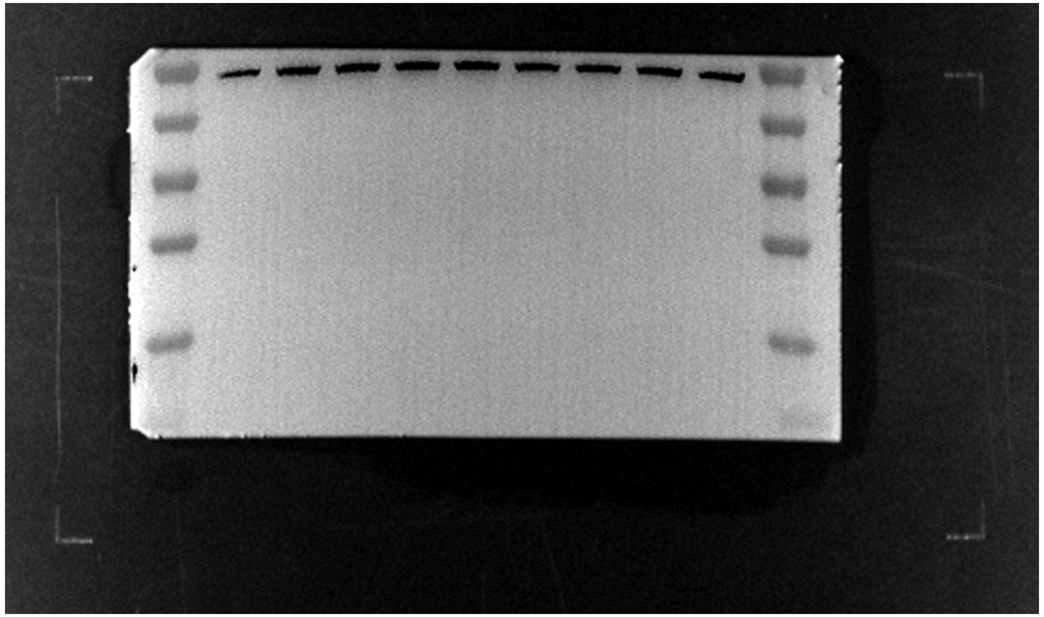

Related to Fig 2B

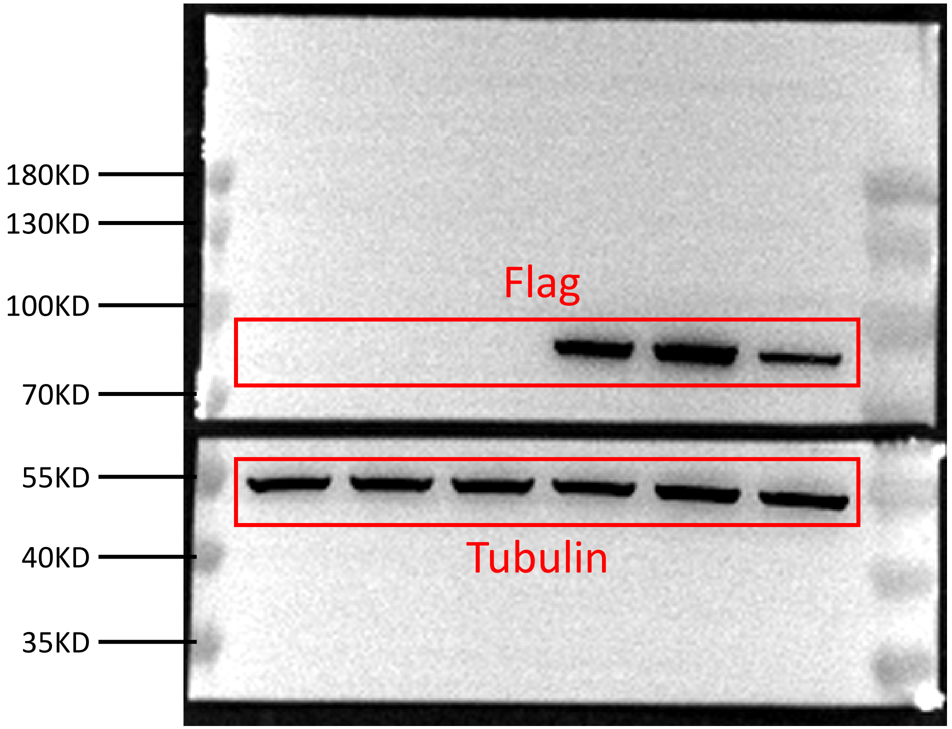

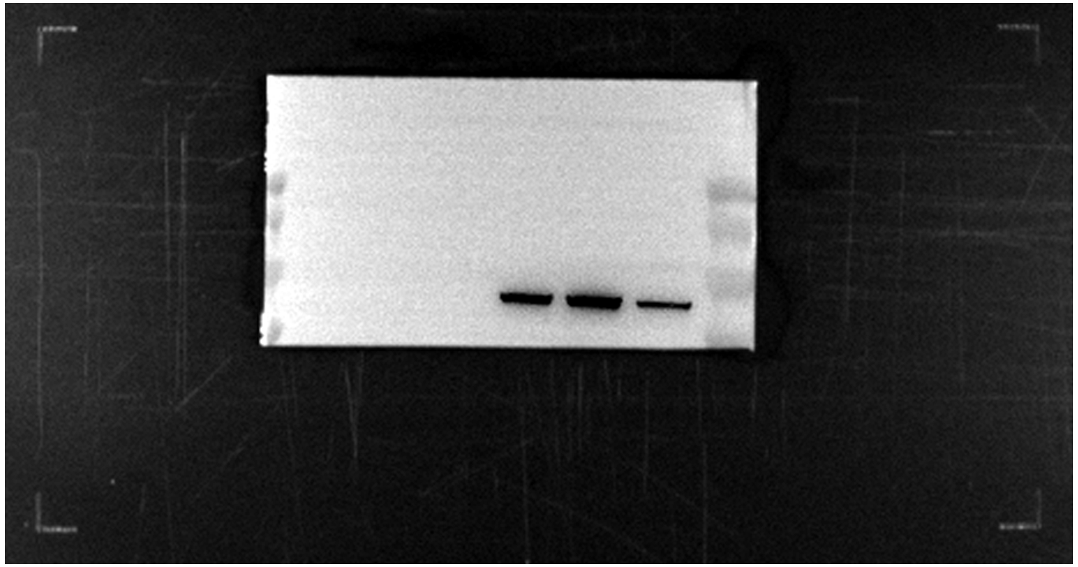

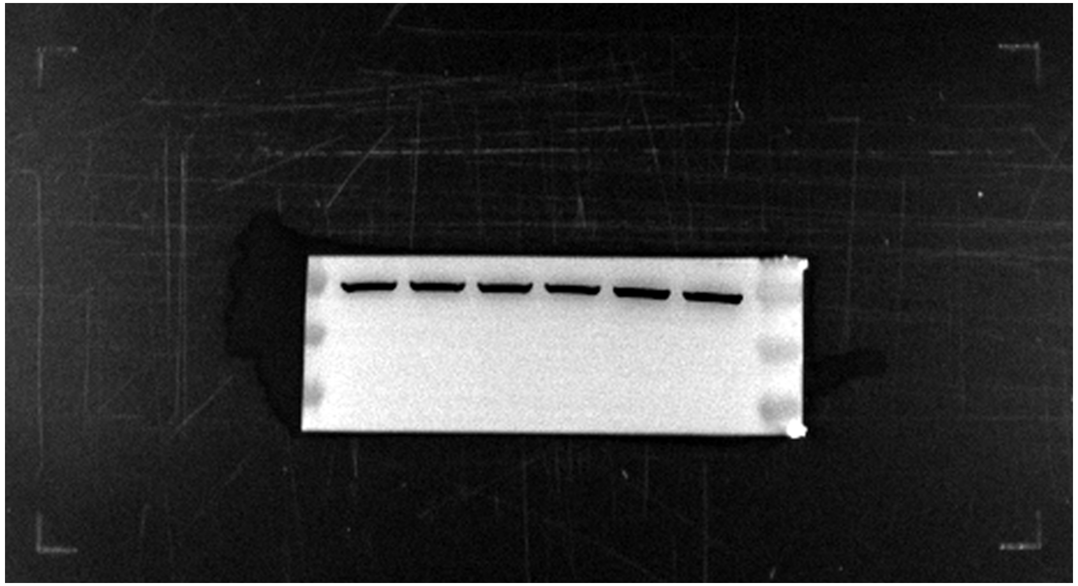

Related to Fig 3C

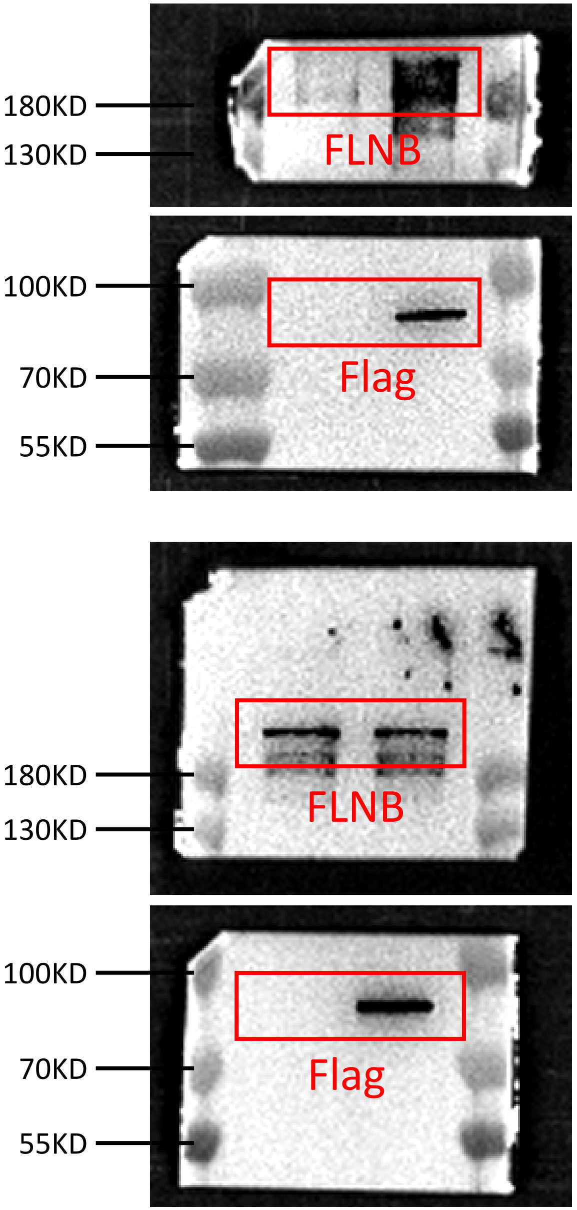

Related to Fig 3D

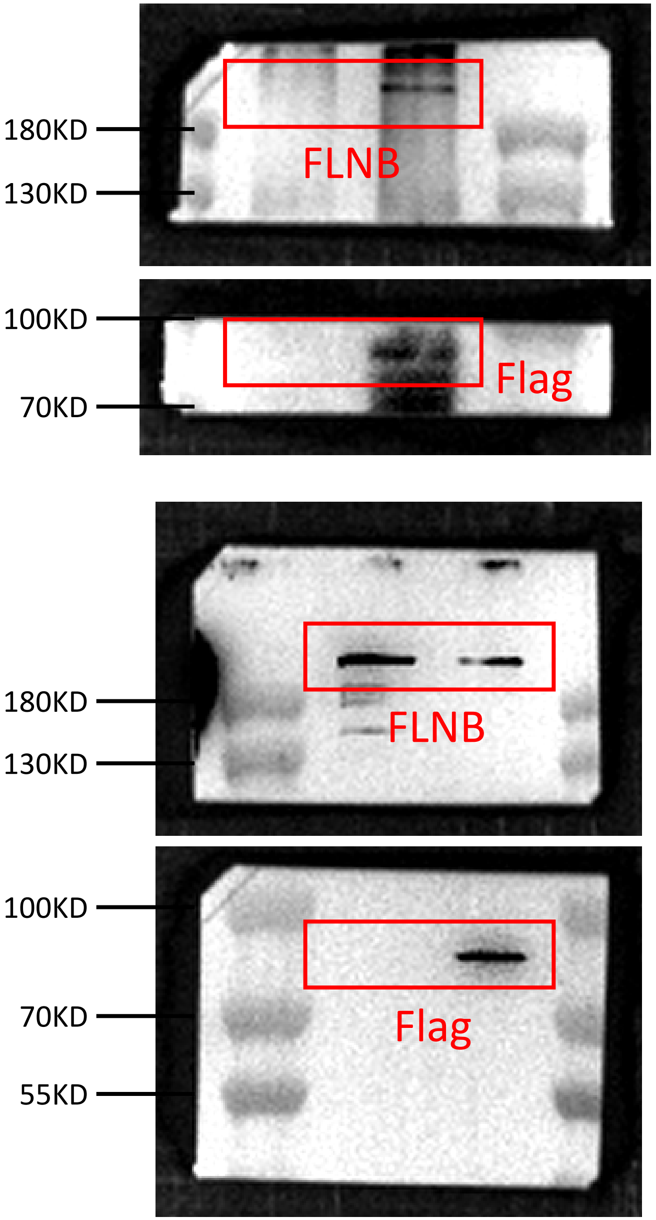

Related to Fig 4A

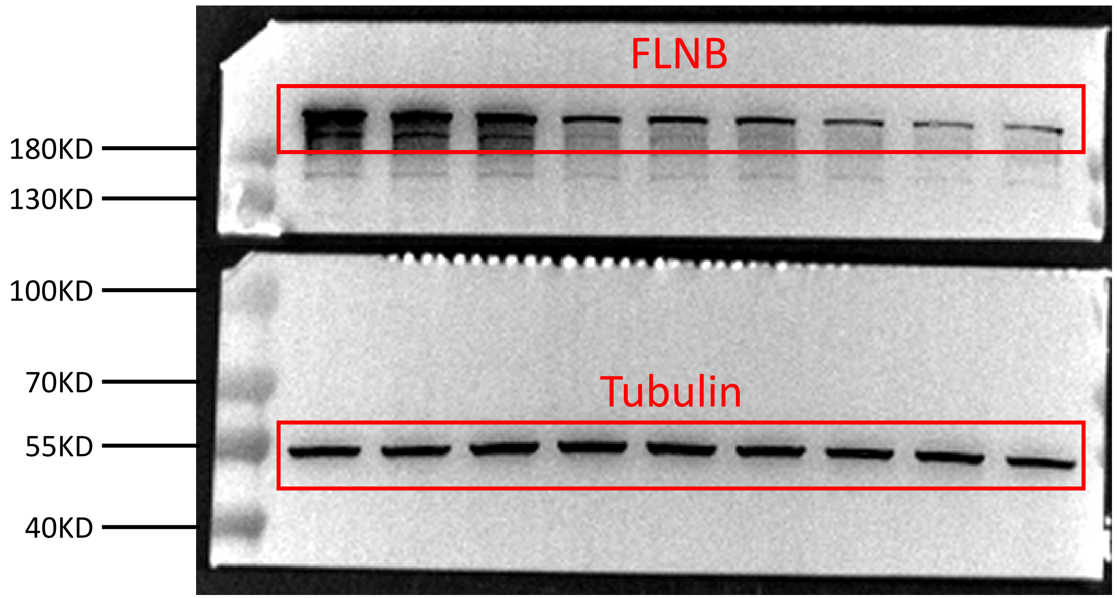

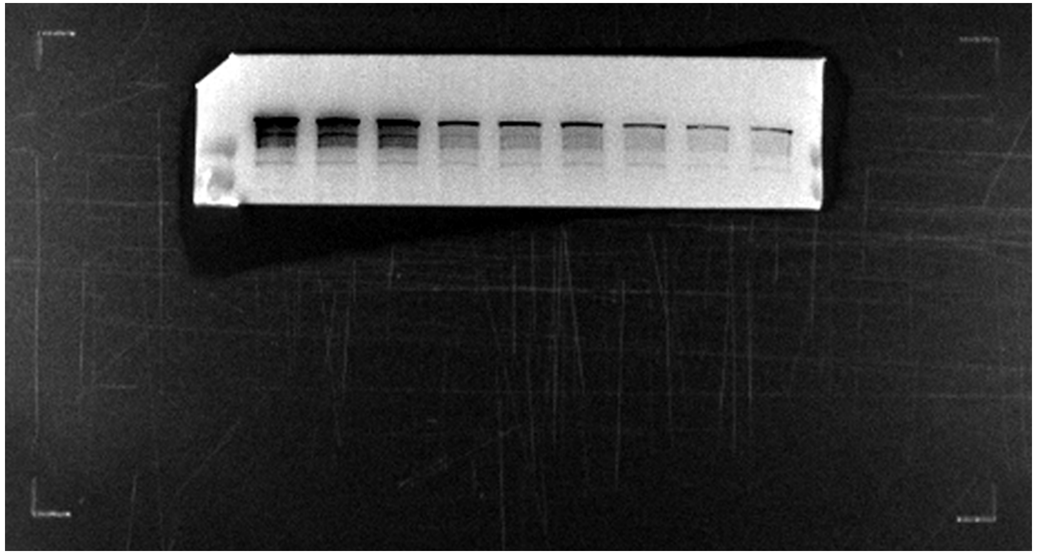

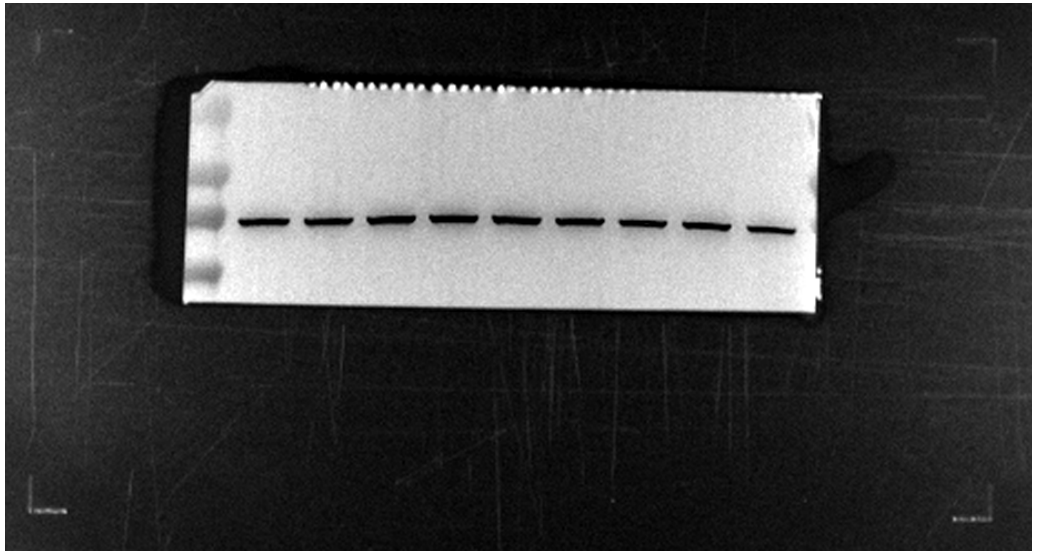

Related to Fig 5A

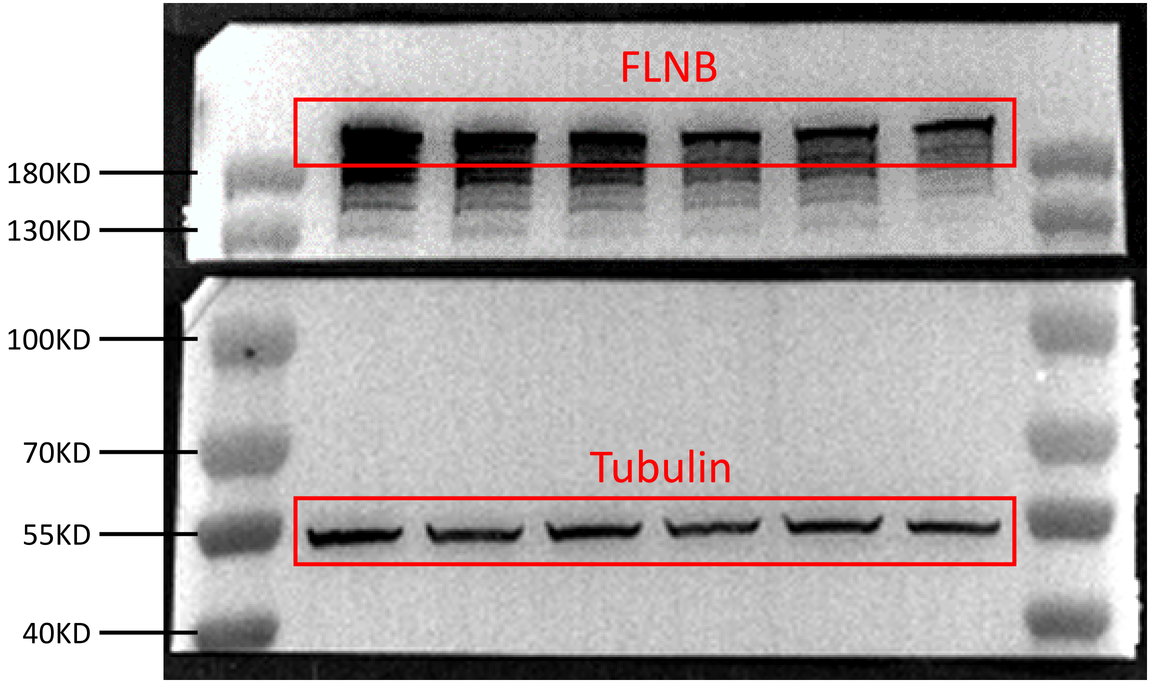

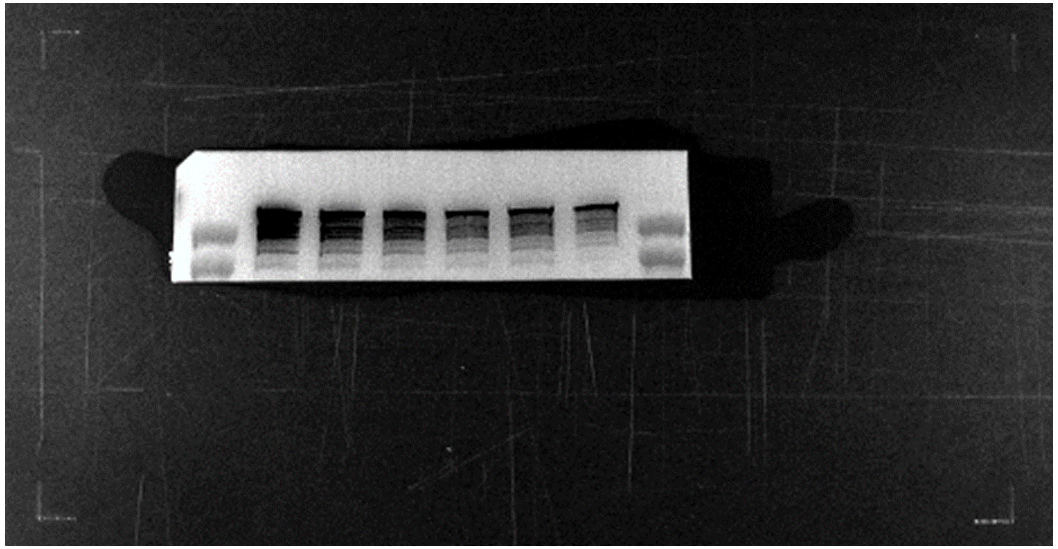

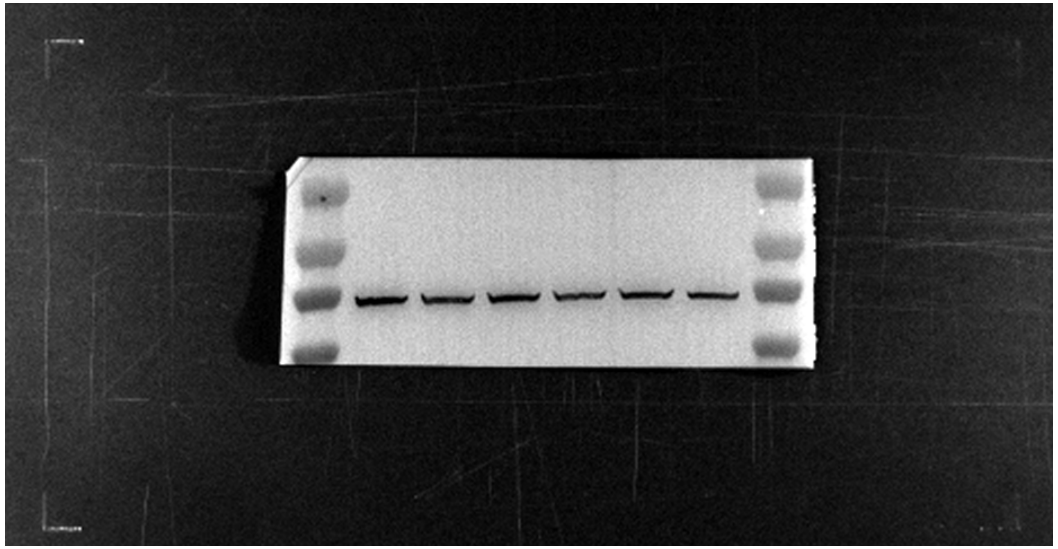

Related to Fig 5C

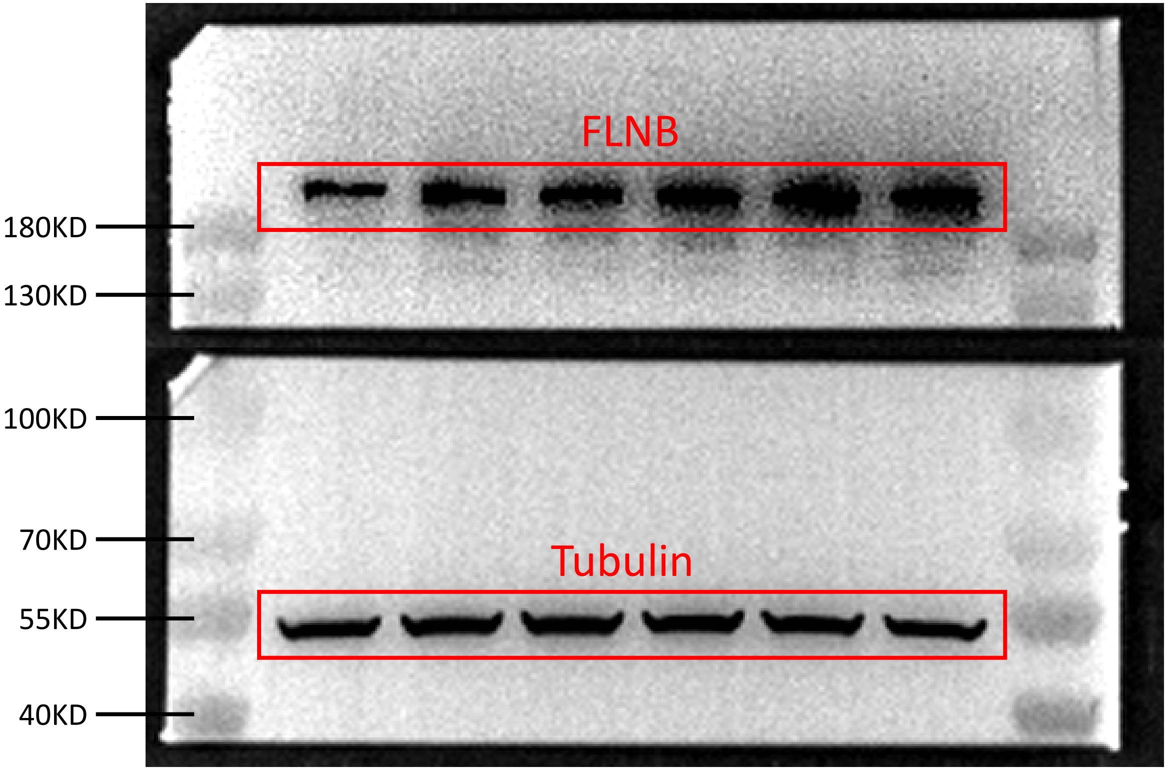

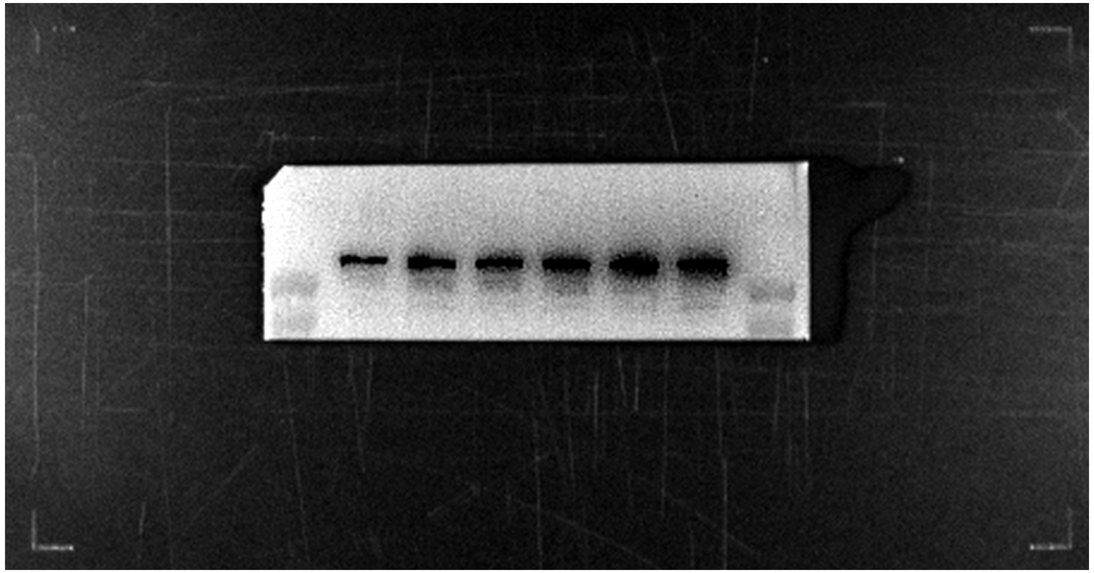

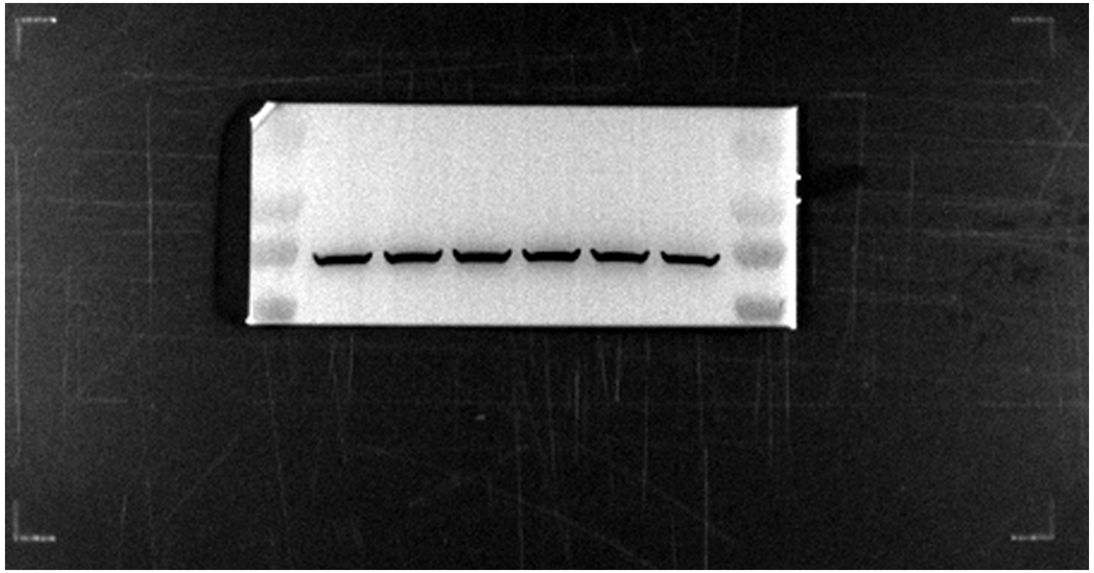

Related to Fig 5E

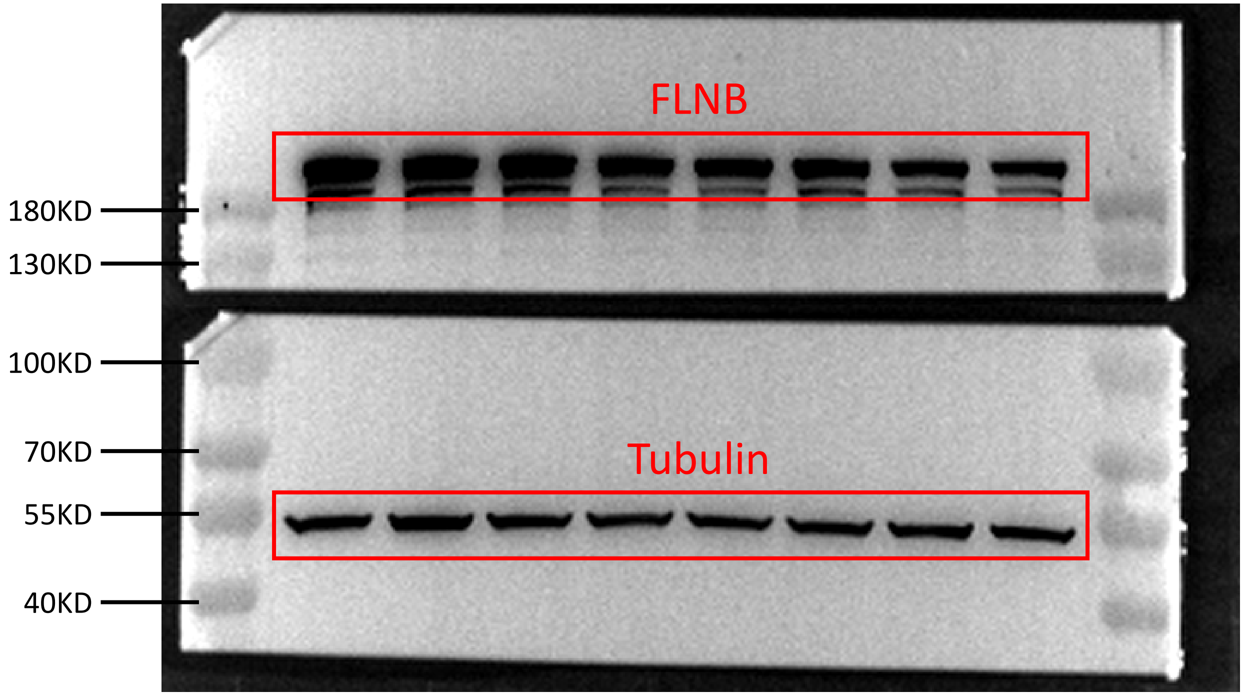

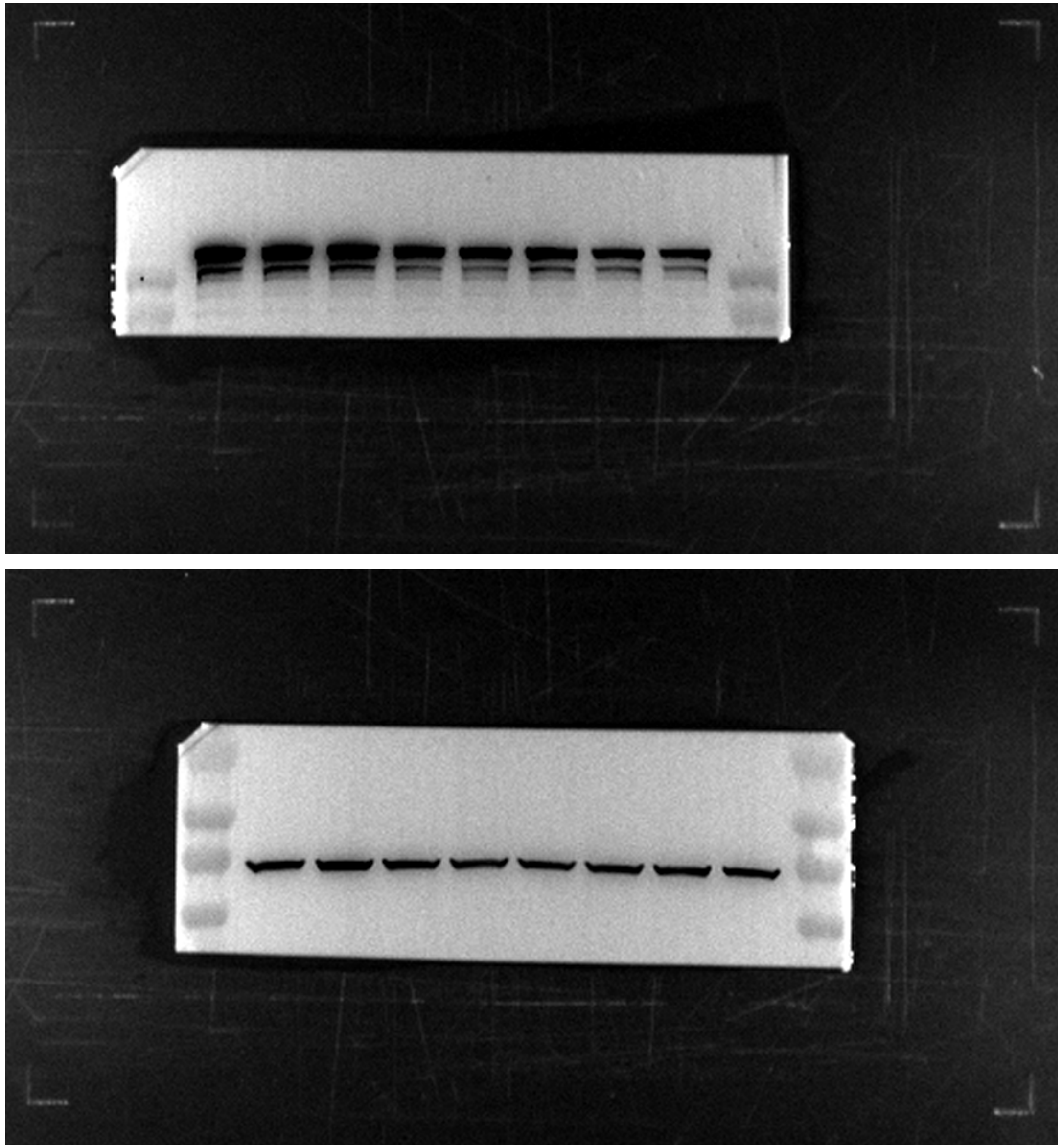

Related to Fig 5G

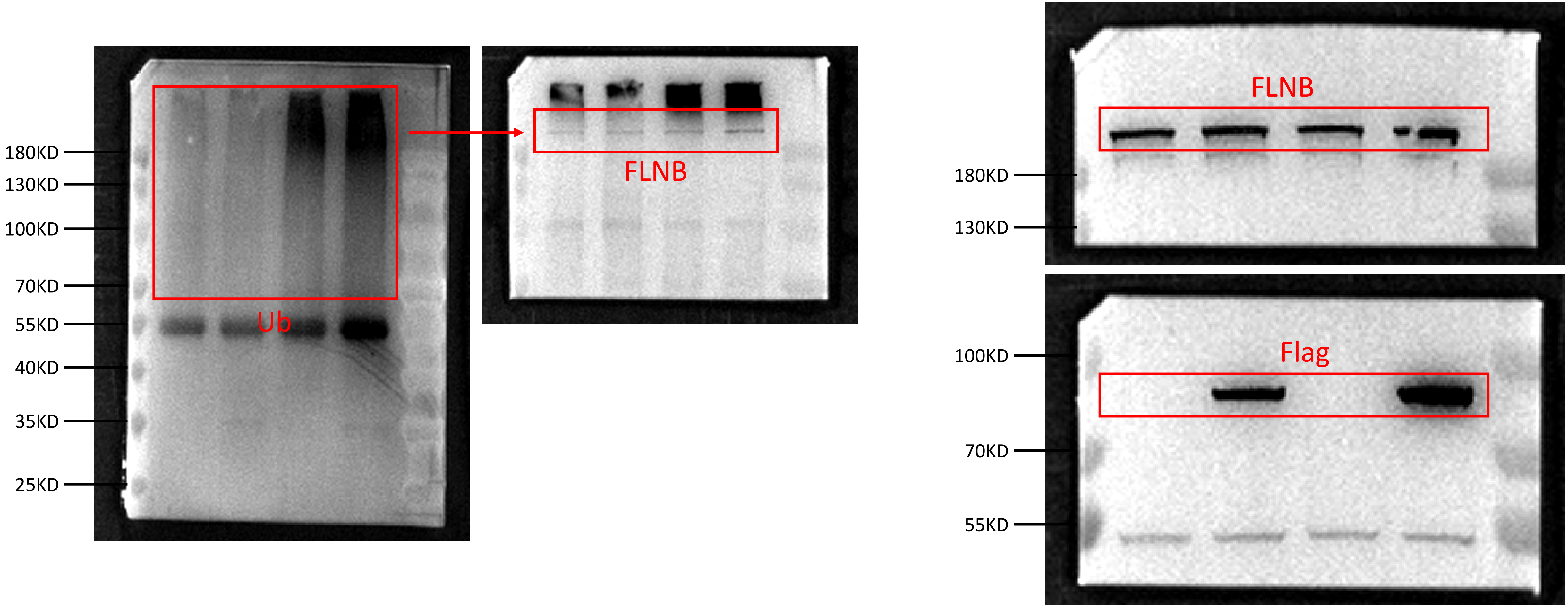

Supplement: S1 raw images — (PDF) [file pone.0327669.s003.pdf]
