# Supplementary material for: MTMR7 regulates human spermatogonial stem cells proliferation and migration via targeting FLNB
Source: PLoS One. 2025 Jul 10;20(7):e0327669. doi: 10.1371/journal.pone.0327669 (PMC12244780; doi:10.1371/journal.pone.0327669)
Supplement: Attachment — Submitted filename: MTMR7 response.docx [file pone.0327669.s005.docx]

***Response***

We sincerely thank the editor and reviewers for their constructive comments and valuable suggestions. We have carefully revised our manuscript according to the comments. Below we provide a detailed point-by-point response to each comment. All changes have been highlighted in the revised manuscript.

***Detailed Responses to Journal requirements***

**1.** Please ensure that your manuscript meets PLOS ONE's style requirements, including those for file naming.

Response: Thank you for your reminder. We have revised the manuscript to meet PLOS ONE’s style requirements, including file naming.

**2.** Please state what role the funders took in the study.  If the funders had no role, please state: "The funders had no role in study design, data collection and analysis, decision to publish, or preparation of the manuscript."

If this statement is not correct you must amend it as needed.

Please include this amended Role of Funder statement in your cover letter; we will change the online submission form on your behalf.

Response: We have amended the Role of Funder statement as requested. The revised statement is included in the cover letter:

*“This work was supported by the National Natural Science Foundation of China (82201762), the Suzhou Gu Su Health Talent Research Project (GSWS2023056), and the Top Talent Support Program for Young and Middle-Aged People of Wuxi Health Committee (BJ2020047). The funders had no role in study design, data collection and analysis, decision to publish, or preparation of the manuscript.”*

**3.** We note that you have provided funding information that is currently declared in your Funding Statement. However, funding information should not appear in the Acknowledgments section or other areas of your manuscript. We will only publish funding information present in the Funding Statement section of the online submission form.

Please remove any funding-related text from the manuscript and let us know how you would like to update your Funding Statement.

Response: Thank you for your reminder. We have removed the funding-related text from the Acknowledgments section as requested, and we have included the revised Funding Statement in the cover letter.

**4.** We note that Figure 6 in your submission contain copyrighted images. All PLOS content is published under the Creative Commons Attribution License (CC BY 4.0), which means that the manuscript, images, and Supporting Information files will be freely available online, and any third party is permitted to access, download, copy, distribute, and use these materials in any way, even commercially, with proper attribution. For more information, see our copyright guidelines: http://journals.plos.org/plosone/s/licenses-and-copyright.

We require you to either (1) present written permission from the copyright holder to publish these figures specifically under the CC BY 4.0 license, or (2) remove the figures from your submission.

Response: We sincerely apologize for this issue. Figure 6 in our manuscript was originally created using Adobe Illustrator, based on concepts and techniques learned from tutorial materials. However, we understand that certain visual elements may appear partially similar to existing materials. We have now revised the figure accordingly and provided the original Adobe Illustrator source files in the supplementary materials (S1_File). If there are still any concerns, please kindly let us know, and we will make further adjustments as needed.

**5.** Please include a separate caption for each figure in your manuscript.

Response: Thank you for your reminder. We have provided a separate caption for each figure in the revised manuscript as requested.

**6.** PLOS ONE now requires that authors provide the original uncropped and unadjusted images underlying all blot or gel results reported in a submission’s figures or Supporting Information files. This policy and the journal’s other requirements for blot/gel reporting and figure preparation are described in detail at https://journals.plos.org/plosone/s/figures#loc-blot-and-gel-reporting-requirements and https://journals.plos.org/plosone/s/figures#loc-preparing-figures-from-image-files. When you submit your revised manuscript, please ensure that your figures adhere fully to these guidelines and provide the original underlying images for all blot or gel data reported in your submission.

Response: We have carefully reviewed the guidelines and ensured that all blot and gel figures in the revised manuscript fully comply with PLOS ONE’s requirements. The original uncropped and unadjusted images underlying all blot and gel results have been provided as Supporting Information files.

***Detailed Responses to Reviewer Comments***

**Reviewer 1**:

1. Introduction:

o Clarify the rationale for focusing on MTMR7 in human SSCs, especially compared to other MTMR family members. Expand on why MTMR7 is particularly relevant in the context of human SSCs.

Response: Thank you for your comment. We have revised the Introduction section to clarify the rationale for focusing on MTMR7 in human SSCs, as compared to other MTMR family members. The revised paragraph is as follows:

*“As a key member of the MTMR family, myotubularin-related protein 7 (MTMR7) differs from other members that primarily dephosphorylate PtdIns3P and PtdIns(3,5)P₂, as it exhibits a stronger catalytic preference for its specific substrate, inositol 1,3-bisphosphate (Ins(1,3)P₂) [25]. This unique enzymatic activity may underlie its distinct biological functions. MTMR7 has been shown to significantly inhibit proliferation across a range of cell types, such as gastric cancer cells, vascular smooth muscle cells, colorectal cancer cells and* *myoblasts [26-29]. In the context of male reproduction, our previous studies demonstrated that MTMR7 is specifically expressed in SSCs in neonatal mouse testes and contributes to the maintenance of cell cycle homeostasis by negatively regulating the PI3K/AKT signaling pathway [30]. These findings suggest that MTMR7 may also serve as a potential biomarker in human SSCs. However, given the phenotypic and regulatory differences between human and mouse SSCs, the precise role and underlying molecular mechanisms of MTMR7 in human SSCs remain to be elucidated.”*

2. Materials and Methods:

o Provide more details on the donor's clinical background (age, health status) for the human testicular tissues.

Response: Thank you for this suggestion. We have revised the *Sample collection* section to provide more details on the donor's clinical background. The revised paragraph is as follows:

*“Human testicular tissues were obtained from a 34-year-old healthy male donor with normal spermatogenesis, whose partner was receiving conventional in vitro fertilization (IVF) treatment at Suzhou Municipal Hospital. The tissue samples were collected during the recruitment period from 30 October 2023 to 1 November 2023. Paraffin sections were prepared by rinsing tissues with phosphate-buffered saline (PBS) and fixing in 4% paraformaldehyde [36, 37]. This study was approved by the Ethics Committee of Suzhou Municipal Hospital (No.2023005) and a written informed consent from the donor was obtained in accordance with the Declaration of Helsinki.”*

o Include additional characterization of the human SSC line (e.g., markers used to confirm its identity as SSCs).

Response: Thanks again for your suggestion. We have revised the *Cell culture* section to include additional characterization of the human SSC line. The revised paragraph is as follows:

*“The human SSC line was generously provided by Professor Zuping He (Hunan Normal University, China). These cells express characteristic SSCs markers, including GPR125, GFRA1, and PLZF, as confirmed by immunocytochemistry [38, 39]. The cells were cultured in DMEM/F-12 medium (Gibco) to support their growth, with additional supplements including 10% fetal bovine serum (FBS, ScienCell, USA) to provide essential nutrients, and 1% penicillin-streptomycin solution to prevent bacterial contamination.”*

o Add data on siRNA efficiency and potential off-target effects for MTMR7 and FLNB knockdown experiments.

Response: Based on your suggestion, we have revised the section on *si-RNA, plasmids, and transfection reagents* by incorporating content addressing siRNA efficiency and potential off-target effects. The updated section now reads as follows:

*“When the cell density reached approximately 70%, overexpression plasmids pcDNA3.1-Flag-MTMR7 and an empty vector (Sangon, Shanghai, China) were introduced into SSCs using the X-treme GENE HP DNA transfection reagent (Mannheim, Germany). For plasmid transfections, 10 μg DNA was used per 10-cm dish and 2 μg per well of a 6-well plate. Separately, siRNAs targeting MTMR7 and FLNB (GenePharma, Suzhou, China) were transfected into SSCs using Lipofectamine 2000 (Invitrogen, USA), with 10 μl siRNAs per well in a 6-well plate [40-42]. To minimize off-target effects, two independent siRNAs were designed for each gene, and only consistent results from both were considered valid. The sequences targeted by the siRNAs were as follows:*

*si-MTMR7-#1: 5′-GCGAUGUGAAUAGAGACUA-3′.*

*si-MTMR7-#2: 5′-CGGCCUAAACUUAAUGCAA-3′.*

*si-FLNB-#1: 5′-GCACGGUCACUGUUAGAUA-3′.*

*si-FLNB-#2: 5′-GAUCGUGUGAUGUCAAAUA-3′.*

*si-NC: 5′-UUCUCCGAACGUGUCACGU-3′.*

*The knockdown efficiency of all siRNAs was validated by qPCR prior to use. Transfection efficiency for both overexpression plasmids and siRNAs was further confirmed by Western blot analysis. The primers used for qPCR are listed below:*

*MTMR7-F: 5′-GTCCGCTTGGTAGATCGAGT-3′.*

*MTMR7-R: 5′-GTAGCGGTTGTTGCCTGTTT-3′.*

*FLNB-F: 5′-TTAAAGGTGACCCGAAGGGTG-3′.*

*FLNB-R: 5′-TGAAGGGACTGCGAGGAATC-3′.*

*18sRNA-F:5′-AAACGGCTACCACATCCAAG-3′.*

*18sRNA-R: 5′-CCTCCAATGGATCCTCGTTA-3′.”*

3. Results:

o Validate the MTMR7-FLNB interaction further using additional techniques like co-localization studies or more Co-IP experiments.

Response: Thank you for the valuable suggestion. We have further validated the interaction between MTMR7 and FLNB by performing three-dimensional (3D) structural modeling and molecular docking analysis using HawkDock. The docking results were visualized with PyMOL. As presented in the revised manuscript (Fig. 3E) and detailed in S2 Table, the 3D model illustrates the potential binding interaction and interface between MTMR7 and FLNB. These findings provide additional support for a direct interaction between MTMR7 and FLNB.

o Include controls in ubiquitination assays to confirm specificity of FLNB ubiquitination.

Response: Thank you very much for this valuable comment. We fully agree that including appropriate controls would further enhance the rigor of our ubiquitination assay. In our current experimental design, we did include certain controls: the EV (empty vector) group was used to exclude non-specific effects of the vector itself, and MG132 treatment confirmed that FLNB ubiquitination is proteasome-dependent. The observed increase in FLNB ubiquitination upon MG132 treatment in the MTMR7 overexpression group supports the specificity of this modification. However, we acknowledge that additional controls such as IP with an unrelated IgG would further strengthen the conclusion. We sincerely appreciate your suggestion and will incorporate such additional controls in future studies to further validate the specificity of target protein ubiquitination.

o Explore downstream effects of β-catenin on SSCs proliferation and migration, such as identifying specific Wnt/β-catenin target genes.

Response: We sincerely appreciate your valuable suggestion regarding the exploration of downstream β-catenin target genes. Due to experimental constraints, we were unable to directly investigate these targets in the current study. However, in response to this helpful comment, we have expanded the *Discussion* section to include relevant literature and potential downstream effectors. We also highlighted the need for future studies to further elucidate their roles within the MTMR7/FLNB/β-catenin regulatory axis. The revised paragraph is as follows:

*“Downstream of β-catenin, target genes such as TCF1, Cyclin D1, Axin2, Lgr5, and c-Myc promote cell cycle progression in both tumor cells and SSCs [65-68]. MMP7, another β-catenin target implicated in hepatocellular carcinoma cell migration [69], may also contribute to SSC motility. In our study, MTMR7-FLNB interaction led to reduced β-catenin levels, which may suppress SSCs proliferation via these downstream effectors. Although their expression was not directly examined, future studies exploring their roles within the MTMR7/FLNB/β-catenin axis may shed light on the molecular mechanisms governing SSCs fate.”*

4. Discussion:

o Address limitations of the study, such as the reliance on a single human SSC line and the lack of in vivo validation.

Response: Thank you for this suggestion. In the revised *Discussion*, we have added statements addressing the limitations of using a single human SSC line and the lack of in vivo validation. The revised paragraph is as follows:

*“Despite uncovering the biological significance of the MTMR7/FLNB/β-catenin axis in SSCs, this study has several limitations. First, the use of a single human SSC line (hSSC-1) may not fully represent the heterogeneity of primary SSCs. Second, the absence of in vivo validation limits our understanding of this axis in a physiological context.”*

o Discuss clinical implications of targeting MTMR7 or FLNB for male infertility treatment, including potential drugs or challenges in translation.

Response: Thank you for this comment. In the revised *Discussion*, we have incorporated a section discussing the potential clinical implications of targeting MTMR7 or FLNB, as well as the challenges in translating these findings into therapeutic strategies. The revised paragraph is as follows:

*“From a clinical perspective, MTMR7 and FLNB may serve as potential diagnostic and therapeutic targets for NOA, particularly in patients with impaired SSCs proliferation. However, several challenges remain, including achieving testis-specific delivery while minimizing systemic effects, and balancing SSCs proliferation and differentiation.”*

5. Figures and Data Presentation:

o Improve labeling in graphs (e.g., Fig 1D, 1E, 2B, 2C) to make time points and conditions clearer.

Response: Thank you for this suggestion. In the revised manuscript, we have updated the labeling of the CCK-8 assay results (Fig 1D, 2B, 4C) to make the time points clearer and easier to interpret. Additionally, we have added scale bars to the colony formation assay images (Fig 1E, 2C, 4D) to improve clarity and consistency in data presentation. We appreciate your advice, which helped us improve the overall quality of our figures.

o Provide a detailed table of LC-MS/MS results in the supplementary materials, listing all identified interacting proteins.

Response: Thank you for this comment. We have provided a detailed table listing all identified MTMR7-interacting proteins from the LC-MS/MS analysis in the supplementary materials (see S1_Table).

6. Supplementary Materials:

o Include full Western blot images, additional validation experiments, and a more comprehensive table of LC-MS/MS results.

Response: We have included the full original Western blot images (S1_raw_images) and the complete LC-MS/MS results (S1_Table) in the supplementary materials as requested.

**Reviewer 2**:

From human testis single cell sequencing data, RNA sequencing data from SSC cells, SSC protein mass spectrometry, and data from the Human protein atlas database, it appears that MTMR7 is a gene with very low or even difficult to detect expression. Is it present in SSC and why did the authors bother to study its role? Please explain the reasons in detail.

Response:

Thank you very much for this important and insightful question. We fully acknowledge that MTMR7 expression appears to be low or even difficult to detect in public datasets such as single-cell RNA-seq or proteomic databases. However, this is in part due to the extreme rarity of SSCs within the human testis, making robust detection of SSC-specific transcripts or proteins inherently challenging in bulk or even single-cell analyses.

In our previous work, we identified MTMR7 in the proteomic profile of neonatal mouse testes and demonstrated that MTMR7 regulates SSCs cell cycle homeostasis via the PI3K/AKT signaling pathway. Building on these findings, we sought to investigate whether MTMR7 might also play a conserved role in human SSCs, given the current lack of understanding regarding human SSCs regulatory mechanisms. Notably, in this study, we observed clear co-localization of MTMR7 with the established SSCs marker PLZF in adult human testis tissue via immunofluorescence staining, providing additional evidence that MTMR7 is indeed expressed in human SSCs at the protein level.

Furthermore, considering the known species differences between mouse and human SSCs in terms of cell types, markers, and phenotypes, we felt it was important to directly explore the potential function of MTMR7 in human SSCs. Our aim was to contribute novel insights into human-specific SSCs biology and address gaps in the current literature. We sincerely appreciate your comment, which has prompted us to further clarify and highlight these considerations in the revised *Introduction* section.

1.Introduction:

The introduction provides a good background on the importance of SSCs in spermatogenesis and male fertility. However, it could benefit from a more detailed discussion on the differences between mouse and human SSCs, particularly regarding the molecular pathways involved. This would help contextualize the significance of studying MTMR7 in human SSCs.

Suggestion: Expand the discussion on the differences in molecular mechanisms between mouse and human SSCs to better justify the need for this study.

Response: Thank you for this valuable suggestion. We fully agree that clarifying the differences between mouse and human SSCs would strengthen the rationale for focusing on human SSCs in this study. Accordingly, we have expanded the *Introduction* section to include a more detailed discussion of these interspecies differences. The revised paragraph is as follows:

*“However, the molecular mechanisms controlling the development of human SSCs remain largely unknown and differ significantly from those in mice [16]. For instance, in addition to the commonly expressed markers PLZF and GPR125, human SSCs uniquely express MAGE-A4 and CD133, while mouse SSCs are marked by CD29, KIT, and POU5F1 [17, 18]. The CXCR4–CXCL12 signaling pathway, essential for SSCs homing to the niche, also differs between species: in humans, CXCL12 is mainly expressed by Leydig cells, whereas in mice, it is predominantly produced by Sertoli cells [19]. These differences underscore the need for species-specific studies to elucidate the regulatory mechanisms governing human SSCs function, which is essential for developing targeted therapies for male infertility.”*

2.Materials and Methods:

The use of si-RNAs and overexpression plasmids is well described, but the authors should provide more information on the efficiency and specificity of these tools. For example, were off-target effects of si-RNAs assessed?

Suggestion: Clearly specify the sample size for each experiment and provide additional details on the efficiency and specificity of the si-RNAs and overexpression plasmids used.

Response: Thanks again for this suggestion. We have revised the *Materials and Methods* section to provide additional details regarding the design, validation, and efficiency of the siRNAs and overexpression plasmids used in our study. We also clarified the measures taken to minimize potential off-target effects. The revised paragraph is as follows:

*“When the cell density reached approximately 70%, overexpression plasmids pcDNA3.1-Flag-MTMR7 and an empty vector (Sangon, Shanghai, China) were introduced into SSCs using the X-treme GENE HP DNA transfection reagent (Mannheim, Germany). For plasmid transfections, 10 μg DNA was used per 10-cm dish and 2 μg per well of a 6-well plate. Separately, siRNAs targeting MTMR7 and FLNB (GenePharma, Suzhou, China) were transfected into SSCs using Lipofectamine 2000 (Invitrogen, USA), with 10 μl siRNAs per well in a 6-well plate [40-42]. To minimize off-target effects, two independent siRNAs were designed for each gene, and only consistent results from both were considered valid. The sequences targeted by the siRNAs were as follows:*

*si-MTMR7-#1: 5′-GCGAUGUGAAUAGAGACUA-3′.*

*si-MTMR7-#2: 5′-CGGCCUAAACUUAAUGCAA-3′.*

*si-FLNB-#1: 5′-GCACGGUCACUGUUAGAUA-3′.*

*si-FLNB-#2: 5′-GAUCGUGUGAUGUCAAAUA-3′.*

*si-NC: 5′-UUCUCCGAACGUGUCACGU-3′.*

*The knockdown efficiency of all siRNAs was validated by qPCR prior to use. Transfection efficiency for both overexpression plasmids and siRNAs was further confirmed by Western blot analysis. The primers used for qPCR are listed below:*

*MTMR7-F: 5′-GTCCGCTTGGTAGATCGAGT-3′.*

*MTMR7-R: 5′-GTAGCGGTTGTTGCCTGTTT-3′.*

*FLNB-F: 5′-TTAAAGGTGACCCGAAGGGTG-3′.*

*FLNB-R: 5′-TGAAGGGACTGCGAGGAATC-3′.*

*18sRNA-F:5′-AAACGGCTACCACATCCAAG-3′.*

*18sRNA-R: 5′-CCTCCAATGGATCCTCGTTA-3′.”*

3.The data on FLNB knockdown and its effects on SSCs proliferation and migration are presented, but the connection to MTMR7's role is not fully explored. The authors should provide a more detailed analysis of how FLNB knockdown mimics the effects of MTMR7 overexpression.

Suggestion: Enhance the clarity of the results by providing a more detailed analysis of the relationship between MTMR7, FLNB, and β-catenin signaling. Consider including additional experiments to further elucidate this relationship.

Response: Thank you for this constructive suggestion. In the revised manuscript, we have strengthened this aspect by providing additional analysis and improving the clarity of the results. First, we included new data from 3D structural modeling and molecular docking (Fig 3E and S2 Table), which support the direct interaction between MTMR7 and FLNB. Furthermore, we have reorganized the structure of the *Results* section to present this regulatory axis in a more logical and coherent manner. We believe these revisions now provide a clearer understanding of how MTMR7 modulates SSCs proliferation and migration through its effects on FLNB and β-catenin.

4.the immunofluorescence images in Figure 5 could be enhanced with more detailed annotations.

Response: We have rearranged the immunofluorescence images in Figure 5 in the *Results* section to improve clarity.

**Reviewer 3**:

1. Introduction Section: The background information on SSCs is insufficient. It would be beneficial to include more details on the biological roles of SSCs and their regulatory mechanisms to provide a stronger foundation for the study.

Response: Thank you for this comment. We have revised the *Introduction* section to include more detailed background information of SSC, providing a stronger foundation for this study. The revised paragraph is as follows:

*“SSCs are adult tissue stem cells located in the testes that are fundamental to spermatogenesis, and their dysfunction can lead to male infertility [7, 8]. SSCs possess dual potential: they can either self-renew to produce new SSCs or differentiate into mature spermatozoa [9]. Maintaining a balance between these processes is crucial for normal spermatogenesis. Earlier research has shown that glial cell line-derived neurotrophic factor (GDNF) is pivotal in controlling both the proliferation and differentiation of mouse SSCs. High concentrations of GDNF promote SSCs proliferation, while low concentrations induce SSCs differentiation [10-12]. Moreover, fibroblast growth factor 2 (FGF2) and retinoic acid (RA) have been recognized as crucial in guiding the developmental decisions of mouse SSCs [13-15]. However, the molecular mechanisms controlling the development of human SSCs remain largely unknown and differ significantly from those in mice [16]. For instance, in addition to the commonly expressed markers PLZF and GPR125, human SSCs uniquely express MAGE-A4 and CD133, while mouse SSCs are marked by CD29, KIT, and POU5F1 [17, 18]. The CXCR4–CXCL12 signaling pathway, essential for SSCs homing to the niche, also differs between species: in humans, CXCL12 is mainly expressed by Leydig cells, whereas in mice, it is predominantly produced by Sertoli cells [19]. These differences underscore the need for species-specific studies to elucidate the regulatory mechanisms governing human SSCs function, which is essential for developing targeted therapies for male infertility.”*

2. Structural Organization: The background information on FLNB, currently included in the Introduction, should be moved to the Discussion section. Since FLNB was identified through subsequent IP experiments, this adjustment would improve the logical flow of the manuscript.

Response: We appreciate your suggestion. The background information on FLNB has been moved from the *Introduction* to the *Discussion* section to improve the logical flow of the manuscript.

Language Simplification:

3. Some sentences are overly verbose and can be streamline:

Line 224: “Fig 1. Reducing MTMR7 levels enhances both the proliferation and migration of human SSCs. ” could be revised to “Fig 1. MTMR7 knockdown promotes proliferation and migration of human SSCs. ”

Line 235: “Western blot analysis confirmed substantial upregulation of MTMR7 protein expression in SSCs transfected with the Flag-MTMR7 plasmid” could be simplified to “Western blot confirmed significant upregulation of MTMR7 in Flag-MTMR7-transfected SSCs. ”

Response: Thank you for the helpful suggestions. We have revised the sentences as suggested to improve clarity and conciseness. The revised sentences are as follows:

Line 248: *“Fig 1. MTMR7 knockdown promotes proliferation and migration of human SSCs. ”*

Line 260: *“Western blot confirmed significant upregulation of MTMR7 in Flag-MTMR7-transfected SSCs. ”*

4. Formal Academic Tone: Some phrases are too informal and should be revised to align with academic writing standards. For example:

Line 416: “These results provide new insights into the regulatory mechanisms affecting human SSCs fate and suggest potential new targets for treating NOA linked to abnormal SSCs development.” could be rephrased as “Our findings elucidate novel regulatory mechanisms governing human SSCs fate and highlight potential therapeutic targets for NOA associated with SSCs dysfunction. ”

Response: Thank you for pointing this out. The sentence in line 416 has been revised to better align with academic writing standards. The revised sentences are as follows:

Line 467: *“Our findings elucidate novel regulatory mechanisms governing human SSCs fate and highlight potential therapeutic targets for NOA associated with SSCs dysfunction. ”*

5. Reference Formatting: Several references are incomplete or incorrectly formatted.

Line 461: “8. .”

Line 520: “24. <10.1007@978-3-030-24108-7.pdf>.”

These should be revised to include complete citation details in the correct format.

Response: Thank you for carefully reviewing the references. We have revised the incomplete and incorrectly formatted references to ensure consistency and accuracy throughout the reference list.
